# Assessment of the multi-sectoral approach to tobacco control policies in South Africa and Togo

**DOI:** 10.1186/s12889-018-5829-3

**Published:** 2018-08-15

**Authors:** Saliyou Sanni, Charles Hongoro, Catherine Ndinda, Jennifer P. Wisdom

**Affiliations:** 1 School of Health Systems and Public Health, Faculty of Heath Sciences, University of Pretoria, Pretoria, South Africa; 2 Population Health, Health Systems and Innovation, Human Sciences Research Council, Pretoria, South Africa; 3 Economic Performance and Development, Human Sciences Research Council, Pretoria, South Africa; 4 City University of New York Graduate School of Public Health and Health Policy, New York, NY USA

**Keywords:** Health policy analysis, Tobacco control, Sub-Saharan Africa

## Abstract

**Background:**

Tobacco use is the world’s leading preventable cause of illness and death and the most important risk factor for non-communicable diseases (NCDs), particularly cardiovascular and chronic respiratory diseases (heart attack, stroke, congestive obstructive pulmonary disease, and lung cancer). Tobacco control is one of the World Health Organization’s “best-buys” interventions to prevent NCDs. This study assessed the use of a multi-sectoral approach (MSA) in developing and implementing tobacco control policies in South Africa and Togo.

**Methods:**

This two-country case study consisted of a document review of tobacco control policies and of key informant interviews (*N* = 56) about the content, context, stakeholders, and strategies employed throughout policy formulation and implementation in South Africa and Togo. To guide our analysis, we used the Comprehensive Framework for Multi-Sectoral Approach to Health Policy, which is built around four major constructs of context, content, stakeholders and strategies.

**Results:**

The findings show that the formulation of tobacco control policies in both countries was driven locally by the political, historical, social and economic contexts, and globally by the adoption WHO Framework Convention on Tobacco Control (FCTC). In both countries, the health department led policy formulation and implementation. The stakeholders involved in South Africa were more diverse, proactive and dynamic than those in Togo, whereas the strategies employed were more straightforward in Togo than in South Africa. The extent of understanding and use of MSA in both countries consisted of an inter-sectoral action for health, whereby the health department strove to collaborate with other sectors within and outside the government. Consequently, information sharing was identified as the main outcome of the interactions between institutions and interest groups within and across three critical sectors of the state, namely the public (government), the private and the civil society.

**Conclusion:**

Tobacco control policies in South Africa and Togo were formulated and implemented from an inter-sectoral approach perspective, which relied heavily on information transfer between stakeholders and less on collaborative problem-solving approach. Incorporation of multiple stakeholders allowed both countries to formulate policies to meet FCTC goals for tobacco control and NCD reduction.

## Background

Tobacco use is the most important risk factor for non-communicable diseases (NCDs), and the world’s largest preventable cause of illness and death. The WHO [1] indicated that tobacco kills nearly seven million people each year, of which more than 600,000 are non-smokers dying from inhalation of environmental tobacco smoke also called environmental tobacco pollution, or second-hand smoke. If no action is taken, tobacco will kill more than 8 million people every year by 2030, with more than 80% of these deaths attributed to inhabitants in low and middle-income countries. There are over 1.1 billion smokers in the world, and cigarette smoking is the most common form of tobacco use. Clearly tobacco use is a widespread and preventable public health problem with substantial impact on low- and middle-income countries.

The WHO-recommended “best-buys” interventions to address tobacco use include protecting people from tobacco smoke and banning it in public places; package warnings about the dangers of tobacco use; enforcing bans on tobacco advertising, promotion and sponsorship; and increasing taxes on tobacco. Formulating and implementing sound tobacco control polices related to these interventions are expected to emerge from interplay between institutions, interests and ideas [2] and to reflect the 2011 United Nations Political Declaration on the Prevention and Control of NCD [3]. This Declaration recognized that prevention must be the cornerstone of the global response to NCD (paragraph 34) and acknowledged the need for a multi-sectoral approach (MSA), including all government levels, to comprehensively and decisively address risk factors and underlying health determinants (paragraph 42). Further, the WHO’s Framework Convention on Tobacco Control, implemented in 2005, was a watershed international treaty that stipulated requirements for signatories to govern the production, sale, distribution, advertisement and taxation of tobacco to reduce its impact on NCDs. Signatories were obligated to update their national policies related to tobacco control.

A multi-sectoral approach (MSA) in the context of health refers to actions of sectors outside the health sector, possibly, but not necessarily, in collaboration with the health sector, on health or health-related outcomes or the determinants of health or health equity [4]. These include actions within and between sectors, at the local, regional, provincial, national, and global levels, needed to influence the social and economic landscape that enables the health and well-being of the population [4]. Engaging in an MSA incorporates three primary approaches [5] to formulating and implementing policy: Inter-Sectoral Action, Healthy Public Policy and Health in all Policies. Inter-Sectoral Action, proposed by the Alma Ata Declaration [6], involves efforts by the health sector to collaborate with other public policy sectors to improve health outcomes. The Ottawa Charter [7] introduced Healthy Public Policy, which involves an explicit concern for health in all areas of public policy through accountability for health impact. Health in all Policies [8], a major theme during the Finnish Presidency of the European Union, is defined as “an approach to public policies across sectors that systematically takes into account the health implications of decisions, seeks synergies, and avoids harmful health impacts in order to improve population health and health equity”.

To assess the extent of understanding and use of MSA to the formulating and implementation of tobacco control policies, based on the three primary approaches to engage in an MSA described above, we developed a Comprehensive Framework for Multi-Sectoral Approach to Health Policy Analysis built around four major constructs of context, content, stakeholders and strategies. Details of this framework are presented in a methodology paper [9]. The study described here was conducted in South Africa, an upper-middle-income and Anglophone country and Togo, a low-income and Francophone one. Tobacco leaf is cultivated, processed, traded, and smoke tobacco products are manufactured in South Africa, whereas Togo mainly hosts tobacco products retailers. The study sought to assess the use of a multi-sectoral approach in the developing and implementing of tobacco control policies in South Africa and Togo. Specific study questions were: (a) How were tobacco control policies formulated and implemented in South Africa and Togo? (b) To what extent was an MSA employed in formulating and implementing these policies? and (c) What were the perceived enablers and barriers to using MSA?

## Methods

This was an independent study inspired by the project on the Analysis of NCD Prevention Policies in Africa, which assessed an MSA for formulating and implementing NCD prevention policies through case studies in five sub-Saharan African countries [10]. Its design consisted of an in-depth investigation of tobacco control policies in a real-life context [11]. The study drew from various sectors selected using a combination of purposive and “snowball” sampling from domestic and international institutions and other interest groups based on their expected role in tobacco control policy formulation and implementation [12]. Study data were collected through document review and key informant interviews. The document review referred to the WHO recommended “best buys” interventions to reduce tobacco use, to assess available legislations and regulations related to the formulating and implementing of tobacco control measures in South Africa and Togo. These policies were researched from government departments, international organisations and non-governmental organisations. The identified ones depicted in Table 1 were assessed with four policy variables, namely, policy content, policy initiator, policy actors and policy instruments [13].

**Table 1:** Policies on tobacco control reviewed in South Africa and Togo

South Africa	Togo
➢ Tobacco Products Control Act, 1993 ➢ Tobacco Products Control Amendment Act, 1999 ➢ Tobacco Products Control Amendment Act, 2007 ➢ Tobacco Products Control Amendment Act, 2008	➢ Law N°2010–017 of 2010 on manufacturing, trade and consumption of cigarette and other tobacco-contained products. ➢ CD^a^ N°2012–046/PR of July 2012: Protecting people from tobacco smoke and banning smoking in public places; ➢ CD^a^ N°2012–047/PR of July 2012: warning about the dangers of tobacco use; ➢ CD^a^ N°2012–071/PR of September 2012: restricting access to retailed tobacco; ➢ CD^a^ N°2012–072/PR of September 2012: enforcing bans on tobacco advertising, promotion and sponsorship;

^a^*CD* Cabinet Decrees

The study participants were key informants who either participated or should have participated in the NCD prevention policy process. These individuals included senior decision makers in the selected sectors such as department or division heads or program managers; heads of NGOs or other actors involved in NCD prevention programs or projects; or heads of private sector institutions or departments and programs within those institutions involved in NCD prevention. To ensure optimal variability across relevant sectors and institutions, the study planned to organise in-depth interviews with up to thirty key informants in each country through a purposive sampling whereby a tracer technique was used to select index key informants and a snowballing technique [12] to identify additional respondents during interviews with index key informants. The key informant interviews were conducted with a semi-structured interview guide. The guide (available upon request) was developed with open- and closed-ended questions focusing on the tobacco control policy context, policy content, actors involved in the process and the implementation status. In addition, data were collected on how an MSA was employed or not, the processes undertaken to ensure that it was followed, the challenges encountered, what worked and what did not work. The operational definition of evidence of an MSA in this study is “involvement of any two or more sectors, one of which must be government.” Sector involvement includes any institutions or interest groups involved in tobacco control policy making; for instance: public sector/government (ministry/cabinet level organization); civil society (NGO, community based organization, faith based organizations); private sector (pharmaceutical company, other industry); research/academic institution (university); and international organizations/bilateral or multilateral. Further, sectoral involvement was categorised as low for just two, as medium when greater than two and below or equal to four, and as high, when greater than four. The interviews were conducted at times and venues mutually agreed upon by the research team and participants. The chosen venues for the interviews were in private places free from distractions and other security risks. All interviews were conducted according to ethical guidelines and most were recorded using a digital recorder. The interviews lasted an average of 60–90 min.

The study used a deductive content analysis approach, which is appropriate for policy-relevant qualitative data. This approach uses an analytical framework featuring key constructs and variables as initial coding categories [14]. Qualitative codes to categorize responses were pre-determined based on the Comprehensive Framework for Multi-Sectoral Approach to Health Policy Analysis depicted in Table 2 and whose details are presented in a methodology paper under review for publication.

**Table 2:** Comprehensive Framework for Multi-Sectoral Approach to Health Policy Analysis

N°	Categories	Elements	Indicators
1	Context	Political context	• Political changes or critical events at the national level that have influenced policy development, • Health sector reforms, fiscal policies among others • Organizational changes (e.g. government structure)
		Timing, Historical/Social factors	• Timeline of policy development • Historical origins of the policy, including what issues it meant to address, and how issue identification has evolved over time. • Other global factors that have influenced policy development and how they influenced it. • Any social factors (e.g. increase in prevalence of NCD)
		Economic context	• Country economic growth • Global and local financial situation and conflicting development agendas
		Technological factors	• Technological factors that have influenced policy development
2	Content	Policy interventions	• Specific NCD prevention policies developed • Which WHO best buy interventions were included • Rationale for developing the policy • Type of interventions (upstream, midstream, or downstream) • Population level coverage of the interventions (universal or targeted) • Implicit or explicit equity goals (improve health of vulnerable groups, reduce health gaps, flatten social gradient)
3	Stakeholders	Institutions (including rules, laws, norms and customs) and interests that led the process of developing health policies	• Government sector/department that led the process • Other sectors that were involved • Levels of government involved (national, local) • Existence of governance structures for multi-sectoral action at different levels (central government, parliament and civil service), their participation in and experiences with these structures. • Civil Society Organisations and private entities involved • Role of sectors involved in formulation (Funding meetings, provision of technical assistance)
		Formulation	• Extent of participation in policy formulation, • Experiences in policy formulation (what went well, and what could have been done differently) • Interests and concerns with the policy process, how these may have influenced their participation and how these were addressed. • Relevant institutions not involved in implementation
		Implementation	• Key sectors/actors involved in the implementation, • Their role in the implementation • Relevant institutions not involved in implementation • Benefits of involving many actors in implementation • Challenges of involving many actors in implementation
4	Strategies	Formulation	• Extent to which the visions held by the health sector, by other sectors and by the ruling party are complementary, comprehensive and coherent • Means of engagement of other sectors, such as consultations, workshops, or meetings. • Patterns of interaction between health and other sectors: • Factors that contributed to successful engagement of other sectors • Benefits of involving different sectors in formulation process • Challenges encountered in the process
		Implementation	• Extent of implementation of the best buys and how implementation is proceeding • Government management styles: - Horizontal integration - Vertical integration - Mix of horizontal and vertical • Any gaps in implementation, the constraints and enabling factors to the implementation process, • Future plans for implementation of the best buys • Mechanisms for monitoring and evaluation
		Funding	• Funding available for implementation of each policy • Sources of funding • Amounts • Funding arrangements such as joint budgeting and delegated financing aimed at addressing supply or demand
		Facilitating factors	• Factors facilitating working together of different sectors
		Hindering factors	• Factors that have hindered working together of different sectors
		Recommendations	• Recommendations and suggestions on how to make multi-sectorality better in the future, • Mechanisms and structures through which multi-sectoral can be enhanced

The transcripts were coded with the elements and indicators of this framework in mind. Nevertheless, the coding left room for other emerging themes outside the framework. Microsoft Excel 2010 software was used to organise data and analyse thematic content. The software was used to collate and consolidate the transcriptions and identify text linked with each content area and key themes. Quotations are reported *verbatim* to illustrate views and concepts and to support conclusions using Giorgi’s phenomenological approach, which focuses on the experiences of participants with shared life experiences [15]. Data analysis and interpretation were iterative.

All study activities were reviewed and overseen by appropriate local ethical review boards in Togo (Ref: 682/2014/MS/CAB/SG/DPLET/CBRS)) and South Africa (HSRC Ref: 2/19/02/114).

## Results

We present the findings of this two-country case study from two standpoints: completeness of the key informant interviews and comparison of the two cases per research questions. To answer the research questions, we referred to the WHO recommended “best buy” interventions to address tobacco use as a major NCD risk factor, and to the major constructs (content, context, stakeholders and strategies) of the study conceptual framework.

### Completeness of the key informant interviews

Out of the 60 planned (30 in each country) interviews, 56 key informants were interviewed to assess the roles of stakeholders in tobacco control policy-making. In South Africa, 26 key informants were interviewed: 14 (54%) of them were male and 12 (46%) were female. In terms of duty stations, nine (35%) were working in Johannesburg, 13 (50%) in Pretoria, two (8%) in Cape Town, one (4%) in Germiston and one (4%) in Tzaneen. In Togo, 30 key informants were interviewed: 25 (83%) of them were male and five (17%) were female. In terms of duty stations, all were working in Lomé, the capital city. Table 3 presents, on matrix used to recruit them, the distribution of the key informants by affiliation in the two study settings.

**Table 3:** Distribution of key informants by affiliation and settings

N°	Institutions and interest groups	Togo		South Africa	
		Index key informant (tracer)	Other respondents (snowballing)	Index key informant (tracer)	Other respondents (snowballing)
1	Health	1	3	1	3
2	Education	1	1	1	1
3	Judiciary	1	1	1	1
4	Legislature	0	1	0	0
5	Law enforcement	1	5	1	1
6	Trade and Transport	2	0	1	0
7	Finance/Treasury	1	2	1	0
8	Agriculture	1	0	1	0
9	The media	1	2	1	1
10	Research institutions	0	0	1	0
11	CSO (Civil society Organisations)	1	3	2	8
12	Tobacco retailers	1	1	0	0
	Sub-total	11	19	12	14
	Grand total	30		26	

### Research question 1: How were tobacco controls policies formulated and implemented in South Africa and Togo?

Regarding policy content, a comparative analysis of the findings from both countries reveals that South Africa and Togo have both passed comprehensive national legislations on tobacco controls, which are almost compliant with the WHO Framework Convention on Tobacco Control [16] they both ratified in 2005. Togo passed one bill for tobacco control in 2010, whereas South Africa required four incremental pieces of legislation between 1993 and 2009. Both countries issued many regulations to put these laws into practice. The extent of implementation of the WHO recommended “best buy” interventions included in the tobacco control policies in both countries is presented in Table 4. In South Africa, there were time-gaps between approval of an act, the president’s assent, the publication in the government gazette and proclamation of commencement. In Togo all these four actions were taken almost concomitantly. In both countries, tax increases on tobacco was the most difficult “best buy” interventions to adopt and implement, and the interviewees supported the challenges of government taxation efforts. For example, an academic official in South Africa indicated that taxation was a significant challenge, including keeping tax increases consistent with inflation. A health department official from this country indicated the challenges of the health department leading the implementation of tobacco taxes when the department does not have specific taxation expertise. In Togo, a Treasury Department official provided an example: when the department sent information to the tobacco industry about proposed tax increases, the tobacco industry responded by providing reports that tobacco control tends to increase illicit trade and that Togo was especially vulnerable because of its porous borders. The Treasury official said, “*That was [the industry’s] way of dissuading us from following what the department of health is saying*.” A law enforcement stakeholder emphasized that, despite challenges from the tobacco industry, stakeholders together were still taking a big-picture view to prevent problems related to tobacco: *“We want to avoid [tobacco] products being dumped in the country.”* Similarly, a community service organization stakeholder stated that the tobacco industry’s “*interferences*” were able to delay implementation of the law requiring health warning pictures on tobacco products for a year.

**Table 4:** Extent of implementation of the “best buys” interventions included in the tobacco control policies in South Africa and Togo

“Best buy” interventions (2014–2016)	Interventions implemented	South Africa	Togo
Tax increases on tobacco	The tax applies to all tobacco products (cigarettes, snuffs, chewing tobacco) (some products = partial)	Yes	Yes
	The tax level during the study	35%	45%
Smoke-free indoor work places and public places	There is a national smoke free policy that covers all public places (some cities or settings = partial)	Yes	Yes
	There are enforced penalties for non-compliance (having penalties but not enforced = partial)	Partial	Partial
Health information and warnings about tobacco	Multiple warnings/images are rotated from time to time, applies to all brands/products	Yes	No
	Large, clear, visible (at least 30% coverage) and legible all brands/all products (if only some of these words are in the legislation = partial)	Yes	Yes
	Health warning includes pictures or pictograms all brands/all products	Yes	Yes
	Include constituents and emissions of tobacco (e.g., how much tar) on all brands/products	Yes	Yes
	In official country language on all brands (only some brands/products = partial)	Yes	Yes
	Required on all tobacco products (if on only some products or brands, partial)	Yes	Yes
Bans on advertising and promotion	Ban advertising, promotion and sponsorship of all tobacco products	Yes	Yes
	Ban for all forms of mass media	Yes	Yes
	Disclosure of expenditure on advertising by industry	Yes	Yes

Regarding the political, historical, social and economic context, findings from this study reveal that the contextual factors in both countries were dissimilar. South Africa is an upper middle-income country with tobacco leaf producers, firms and tobacco manufacturing companies, while Togo is a low-income country, hosting only some tobacco retailers. In South Africa, prior to 1993, the political, historical and social contexts of the tobacco control policy were characterised by a lack of government interest because the tobacco industry was dominated by white, Afrikaans-speaking South Africans with close ties to the apartheid government. The historical and social contexts began to turn in favour of public health measures for controlling tobacco use in South Africa in 1988 when a special issue on tobacco was published in the *South African Medical Journal* to coincide with the first World No Tobacco Day. The issue addressed the health and economic impacts of tobacco use and advocated the need for tobacco control policy in South Africa. The collapse of apartheid in the early 1990s and the new African National Congress (ANC)-led government provided a window of opportunity from the political stream for the tobacco control policy-making process [17]. In addition, an academic key stakeholder noted that a 1993 international conference on tobacco use and its control in Africa held in Harare, Zimbabwe, was attended by scientist and minister of health Dr. Nkosazana Dlamini-Zuma who gave the opening speech in her capacity as a representative of the ANC. The stakeholder noted her presence and speech sent a strong message to the conference participants. The change in political landscape continued with the first democratic elections in 1994, which brought into power Nelson Mandela, enormously helped tobacco control cause in South Africa. Indeed, the ANC, the new ruling party, had no alliance with the tobacco industry and had much stronger commitment to an effective tobacco control policy since Nelson Mandela had consistently voiced his strong support for anti-smoking legislation and was on record as having called for a “world free of tobacco [17].” The consistent political support since 1994 enabled hectic but successful development and implementation of the tobacco control policies as described above. A key stakeholder at a civil society organization also mentioned Dr. Dlamini-Zuma as being a specific powerful voice who supported tobacco control policies despite pressure from the tobacco industry in South Africa and Afrikaans-speaking whites, who protected the tobacco industry, to maintain the status quo. Likewise, Dr. Dlamini-Zuma was also on record for requiring smoke-free cabinet meetings [17]. The stakeholder mentioned above indicated that the ability of the health office to use data to identify impacts was particularly useful (e.g., countering tobacco companies’ warnings about the policies resulting in job loss by showing minimal impact on jobs).

In Togo, unlike in South Africa, tobacco control was not an issue of “high politics,” so it was relatively easy to merge the problem, policy and politics streams and convince the government to act. A law enforcement stakeholder articulated the approach from his perspective: “*Togo has ratified a number of conventions and to abide by these conventions, there was a need to align national laws and regulations with the global commitments*.” He went on to discuss the challenge of illicit use of Togo’s ports by other countries to evade taxes and provide tobacco on the black market. Increasing awareness of this challenge led to a ministerial order to prohibit the practice.

Considering stakeholders, actors from the critical three sectors of the state—namely, public sector (government), private sector and civil society [18]—were involved in policy-making on tobacco control in both countries as depicted in Table 2. The government, through the Department of Health, led the process in both countries and had support from civil society organisations to overcome barriers from the private sector. However, involvement and support of stakeholders from the research institutions and civil society organisations were more diverse, proactive and dynamic in South Africa than in Togo. Indeed, although the health department led the process in both countries, the research institutions and civil society organisations played a much greater role in South Africa than Togo. In both countries, the justice, law enforcement and media sectors, who considered themselves as key stakeholders, felt left out in the policy formulation process, especially when they were later called to act in policy implementation. For example, in South Africa, a police official said, “*It’s a pity we were not involved,*” and both police and justice officials suggested that their involvement could have strengthened the policies by clarifying policies for monitoring and penalties for those to do not follow the implementation guidelines. Similarly, a media official suggested the media industry could have also offered more in the formulation process instead of being involved. In Togo, a justice official said, “*I was not involved in the formulation of the law passed. I do not know [ …] if the Justice [department] had shared their viewpoints about the infractions mentioned in the law. Thus, I think it is during the implementation of this law that the justice is approached just to implement the law.*” Similarly, a law enforcement official said, “*It is after the law was passed that the national anti-drug committee were approached to see how the law enforcement can contribute to its implementation. […] If I were involved in the formulation of the law I would have included some of the aspects of the fight against tobacco in the fight against drugs. I would have also shared my experience on the fight against drugs*.”

In both countries, other sectors mainly involved in the implementation also stated that they should have been involved at the formulation stage.

The study found the strategies employed in tobacco control policy-making more straightforward in Togo than in South Africa. Indeed, in a low political context, with readily available evidence provided mainly by the WHO to the Health Department, policy-makers in Togo managed to overcome resistance from the representatives of the tobacco and hospitality industry and persuade the Parliament to pass a tobacco control law almost compliant with the WHO FCTC^16^: health warning pictures were left out of the law in Togo. Conversely, the high political context in South Africa with stakeholders who have vested interests in blocking or weakening the tobacco control policies, the policy-makers—led by the Department of Health and supported by the research institutions and the civil society organisations—used a combination of science, evidence and politics, including strong activism to succeed. Otherwise, in both countries, the health department led the process and engaged other sectors through consultations, workshops or meetings, mostly funded by the partners, particularly in Togo. For instance, in Togo, a health department stakeholder described the department’s role as a “*peacemaker*.” He said, *“It is us who triggered the process and we produced, within a team with legal experts of the department of health, a draft which we shared with other departments of the government to check if the content of the draft is agreeable to them;…the department of health is the one that coordinates, monitors and evaluates. The other departments check the applicability in their domains.”*

Further, in both countries the interaction between the health department and other sectors during policy formulation and implementation consisted mainly on information-sharing and rarely went further to cooperation, coordination or integration. Lastly, in both countries, no funding was earmarked or internally designated to implement tobacco control measures and most of the catalytic funds came from donors.

### Research question 2: To what extent was MSA employed in policies’ formulation and implementation in South Africa and Togo?

Table 5 presents the distribution of sectors’ involvement in policy formulation and implementation in South Africa and Togo. Data depicted in that table indicate that, in both countries, the MSA was employed to a great extent in that many sectors (more than four) were involved in formulating and implementing tobacco control policies. In both countries more sectors were involved in the implementation than in the formulation stage, and civil society organisations were highly involved particularly in South Africa.

**Table 5:** Sectors’ involvement in tobacco control policy formulation and implementation by Sector in South Africa and Togo

South Africa				Togo			
Sectors	Organisa-tions^a^	Formu-lation^b^	Implemen-tation^b^	Sectors	Organisations^a^	Formulation^b^	Implementation^b^
Health	4	2	4	Health	4	2	2
Education	2	0	2	Education	2	1	2
Judiciary	2	0	2	Judiciary	2	0	2
Legislature	NA	NA	NA	Legislature	1	1	0
Law enforcement	2	0	2	Law enforcement	6	0	6
Trade & transport	1	0	1	Trade & transport	2	1	1
Finance/treasury	1	0	1	Finance/treasury	3	0	3
Agriculture	1	0	0	Agriculture	1	0	0
The media	2	0	2	The media	3	2	3
Research Institutions	1	1	1	Research Institutions	NA	NA	NA
Civil society organisation	10	6	10	Civil society organisation	3	2	1
Tobacco industry	NA	NA	NA		3	2	2
Total	26			Total	30		

^a^number of representatives of organisations (institutions or interest groups) interviewed within sectors

^b^number of organisations within sectors involved in policy formulation or implementation

### Research question 3: What are stakeholders’ perceived enablers and barriers to the use of the MSA in South Africa and Togo?

Table 6 shows that the facilitators and barriers to the MSA were similar in nature, but were not of equal importance in both countries. Indeed, in South Africa, local expertise through several scientific publications from research and academic institutions and a strong political will initially from the post-apartheid government are the most important facilitating factors, both at the policy formulation and implementation stages, and they are higher than the ratification of the WHO FCTC. Conversely, in Togo, the WHO FCTC is the leading facilitator of the MSA in the tobacco control policy-making process. In both countries, the tobacco industries have been the main barriers to formulating and implementing tobacco control policies, but they are stronger in South Africa than in Togo because of their noticeable contribution to country revenues and their ties to the ruling power, particularly during the apartheid era. In Togo, a health official stakeholder described his view on the tobacco industry, “*The problem is that the tobacco industry did not want any policy on tobacco control and tried their best to block the law. But the Togo’s authorities chose the health of the population and the law was passed; it is the tobacco industry that corrupts and precludes people from fulfilling their mission.”* An education official stakeholder provided this strong perspective on the conflicting issues, “*In Togo, foreigners smoke a lot; however, we need them for country development. And more, there is democracy, freedom of movement of people and goods which make difficult to control the use of tobacco in the countries, not only in Togo. Now there is pressure from the tobacco industry. They are powerful people, clever, very strong who easily manage to corrupt.*”

**Table 6:** Facilitators and barriers to the use of MSA in tobacco control policies in South Africa and Togo, in decreasing order of importance

Policy stages	South Africa		Togo	
	Facilitators	Barriers	Facilitators	Barriers
Formulation	• Local expertise: evidence from research that supports legislation • Political will: public participation requirement in policy formulation • Nucleus group -initiates and drives the policy formulation process- critical in ensuring that content and process issues are covered in policy drafting • A central co-ordination point -workshops and drafting sessions are strategically convened to include most stakeholders • Ratification of the WHO FCTC in April 2005 • Personal motivation of the stakeholders • Donor catalytic funding	• The tobacco industry • Weakness in coordination: patterns of interaction between health and other sectors limited to information sharing • Different stakeholder expectations • Inadequate funding and overdependence on donors • Lack of participation of women groups	• Ratification of the WHO FCTC in November 2005 • Political will • Availability of local expertise • Donor catalytic funding • Personal motivation of the stakeholders	• Weakness in coordination: patterns of interaction between health and other sectors limited to information sharing • The tobacco industry • Different expectations • Inadequate funding and overdependence on donors • Lack of participation of women groups
Implementation	• Local expertise • Political will • Personal motivation of the stakeholders • Ratification of the WHO FCTC in April 2005	• The tobacco industry • Government management styles: more vertical than horizontal integration • Public participation: MSA is a requirement in policy-making but not in policy implementation. Nothing compels stakeholders to collaborate in implementing the tobacco control policy and other NCD policies in general • Different stakeholder expectations • Inadequate funding and overdependence on donors • Lack of participation of women groups	• Ratification of the WHO FCTC in November 2005 • Political will • Local expertise • donor catalytic funding • Personal motivation of the stakeholders	• The tobacco industry • Government management styles: more vertical than horizontal integration • Different expectations • Inadequate funding and overdependence on donors • Lack of participation of women groups

## Discussion

The concept of four-by-four refers to the fact that the four major NCDs—namely, cardiovascular diseases, cancers, chronic respiratory diseases and diabetes—share four major behavioural risk factors, which are tobacco use, unhealthy diet, physical inactivity and harmful alcohol use.

The WHO [19] postulated that to be effective, NCD prevention policies should focus on the four major modifiable risk factors of the four major diseases, be formulated and implemented through an MSA and be analysed from a political and organisational perspective of health policy analysis. The study findings are discussed from three standpoints: (a) soundness of the formulation and level of implementation of tobacco control policies in South Africa and Togo, (b) effectiveness of the use of MSA; and (c) extent of its understanding and use in these countries.

### Soundness of the formulation and level implementation of tobacco control policies in South Africa and Togo

Results of the data analyses from document review and key-informant interviews showed evidence of formulation and implementation in South Africa and Togo of policies related to the WHO recommended “best buy” interventions to address tobacco use as a major NCD risk factor. The formulation of such tobacco control policies in both countries was driven locally by the political, historical, social and economic contexts, and globally by the adoption of the WHO Framework Convention Tobacco Control. The stakeholders involved were more diverse, proactive and dynamic in South Africa than in Togo, whereas the strategies employed were more straightforward in Togo than South Africa. The findings indicated that the process was led, in both countries, by the health department instead of the cabinet or a supra-departmental commission, which did not permit significant interactions among other departments of the government (whole of government) that have a stake in tobacco control policy-making. In the absence of such a whole of government approach [20], information sharing was identified as the main outcomes of the interaction between institutions and interest groups within and across three critical sectors of the state, namely public sector (government), private sector and civil society. This insufficient level of interactions contributed to the low implementation of tobacco control policies in both countries.

These findings are similar to those from the case studies on tobacco control in Cameroon, Kenya, Malawi and Nigeria in the sense that different countries are at varying stages on the continuum of tobacco control policy formulation and implementation [10]. Indeed, South Africa, Kenya, Togo and Nigeria have fully developed policies signed into law, whereas Cameroon has several piecemeal policies not yet signed into law and Malawi has not even ratified the Framework Convention on Tobacco Control (FCTC).

### Effectiveness of the use of MSA in tobacco control policies in South Africa and Togo

Applying the study operational definition of MSA to data generated from document review and key-informants interviews showed that: (a) the MSA was highly used in formulating and implementing tobacco control policies in South Africa and Togo; (b) more sectors were involved at the implementation than at the formulation stage; and (c) the civil society organisations were highly involved, although more in South Africa than Togo. However, the study findings indicate variety in the extent to which sectors were at least nominally involved in policy formulation and implementation, and participants generally stated that MSA was very useful in understanding stakeholder perspectives and formulating actionable plans that address multiple contingencies. Further, since the findings are limited in a retrospective study design, it was not possible to assess the extent to which each participated (e.g., attending a single meeting vs. actively drafting document). It would be useful for the measurement of MSA to provide a baseline for what “counts” as involvement. Therefore, the study recommends that merely attending a meeting is not likely enough to contribute meaningfully to policy formulation and that some sort of “active” participation should be measured (e.g., providing testimony, information, drafting, or review).

The coding scheme for low, medium, and high MSA provided an overview of the variety of MSA in formulating and implementing tobacco control policies in South Africa and Togo, but the study was not able to assess the relationship between the number of sectors involved and the country’s effectiveness and timeliness in policy formulation. The study findings did not also enable to comment on the impact of single vs. multiple stakeholders within one sector; for example, the health sector is considered to be participating if only the health ministry is involved, but the health sector could have greater impact if sector participation includes the health ministry, non-governmental organizations related to health, and health researchers compared to health ministry participation only. It would be useful to establish a measurement tool that can be used across types of policy formulation that accurately assesses (a) the extent to which stakeholders were “actively” participating in policy formulation, (b) the impact of multiple organizations within sector compared to a single organization within a sector, and (c) the number or kind of sectors (e.g., as many as possible, specific key sectors, a minimum number of sector) that are most highly associated with effective and timely policy formulation and implementation.

### Extent of understanding and use of MSA in tobacco control policies in South Africa and Togo

As the first focus of global response to the challenge of NCDs, the 2011 United Nations High Level Meeting Political Declaration “recognizes that the rising prevalence, morbidity and mortality of NCDs worldwide can be largely prevented and controlled through collective and multi-sectoral action by all member states and other relevant stakeholders…” The WHO 2013–2020 Action Plan for the Prevention and Control of NCDs reiterated the MSA as cornerstone for NCD prevention at the population level. This plan also emphasised some “best buy” interventions for NCD prevention, including measures to reduce the four common risk factors of tobacco use, unhealthy diet, physical inactivity and the harmful use of alcohol. Moreover, these attempts would deliver the greatest benefit in reducing population-level risks in a cost-effective manner.^19^ The WHO (1998) has defined the Inter-Sectoral Action for Health approach as “a recognised relationship between part or parts of the health sector with part or parts of another sector which has been formed to take action on an issue to achieve health outcomes (or intermediate health outcomes) in a way that is more effective, efficient or sustainable than could be achieved by the health sector acting alone” [21]. In the above mentioned UN documents, the terms multi-sectoral and inter-sectoral are used interchangeably, and it is within this context, that WHO Member States, including South Africa and Togo, signed and started the implementation of the global commitments related to NCDs prevention and control.

In reference to the three policy strategies proposed by Kickbusch [22] to synthetize the various interpretations and scope of the multi-sectoral approach in the context of health described in the introduction, the extent of understanding and use of the MSA in formulating and implementing tobacco control policies in South Africa and Togo can be characterized as an Inter-Sectoral Action for Health. Indeed, because, in both countries, the process was led by the health department instead of the cabinet or a supra-departmental commission, it did not permit significant interactions among other governments departments (whole of government) that have a stake in tobacco control policy-making. On the one hand, Inter-Sectoral Action for Health is a narrow and issue-centred approach to the MSA, which aims to integrate a specific health concern into other relevant sectors’ policies, strategies and plan, in line with the 1998 WHO definition mentioned above. On the other hand, it is at the beginning of a continuum of degrees of policy integration [23] based on Kickbusch’s MSA typology. Therefore, the Inter-Sectoral Action for Health understanding and use of a multi-sectoral approach to tobacco control policies in South Africa and Togo can be further characterized as minimal.

## Conclusion

We found from this two-country case study that: (a) South Africa and Togo have used a multi-sectoral approach to formulate and implement comprehensive national policies on tobacco control, which are almost compliant with the WHO Framework Convention on Tobacco Control [16] they both ratified in 2005; (b) the process was led, in both countries, by the health department instead of the cabinet or a supra-departmental commission, which did not permit significant interactions among other government departments with a stake in tobacco control policy-making; (c) only multi-sectoral, whole-of-government and whole-of-society responses are appropriate to simultaneously address the NCD risk factors, particularly tobacco use, and their underlying determinants such as residence, education, material well-being and access to health to health care. These findings can contribute to the 2030 United Nations’ agenda for sustainable developments [24] that calls for reducing premature mortality from NCDs by one-third, strengthening the implementation of the WHO FCTC in all countries and achieving universal health coverage, including financial risk protection, access to quality essential health care. Further, the study’s findings about the effectiveness, but narrow and minimal understanding and use of MSA in the context of health (based on Kickbusch’s typology) can contribute to the understanding and implementation of the WHO’s recommendation for multi-sectoral action for health in formulating and implementing non-communicable disease prevention policies in the context of United Nations and WHO goals for global health.

Although this two-country study was not able to address all the challenges related to the variety in the extent to which sectors were at least nominally involved in policy formulation and implementation, it does provide a solid first step in baseline information about MSA and in clarifying measurement issues that can be addressed by future research. For instance there is an urgent need to address MSA measurement issues, including defining minimal involvement to be considered “active,” the impact of multiple organizations within sector compared to a single organization within a sector, and the number or kind of sectors that are most highly associated with effective and timely policy formulation and implementation. Note accurate measurement includes a reliable and valid outcome measure of policy effectiveness, timeliness, comprehensiveness, or impact.

## Abbreviations

ANPPA: Analysis of Non-communicable Diseases Prevention Policies in Africa
FCTC: Framework Convention on Tobacco Control
MSA: Multi-Sectoral Approach
NCD: Non-Communicable Diseases
NGO: Non-Governmental Organisations
WHO: World Health Organisation
